# Accumulation of Biomass and Mineral Elements with Calendar Time by Corn: Application of the Expanded Growth Model

**DOI:** 10.1371/journal.pone.0028515

**Published:** 2011-12-14

**Authors:** Allen R. Overman, Richard V. Scholtz

**Affiliations:** Agricultural & Biological Engineering Department, University of Florida, Gainesville, Florida, United States of America; United States Department of Agriculture, United States of America

## Abstract

The expanded growth model is developed to describe accumulation of plant biomass (Mg ha^−1^) and mineral elements (kg ha^−1^) in with calendar time (wk). Accumulation of plant biomass with calendar time occurs as a result of photosynthesis for green land-based plants. A corresponding accumulation of mineral elements such as nitrogen, phosphorus, and potassium occurs from the soil through plant roots. In this analysis, the expanded growth model is tested against high quality, published data on corn (Zea mays L.) growth. Data from a field study in South Carolina was used to evaluate the application of the model, where the planting time of April 2 in the field study maximized the capture of solar energy for biomass production. The growth model predicts a simple linear relationship between biomass yield and the growth quantifier, which is confirmed with the data. The growth quantifier incorporates the unit processes of distribution of solar energy which drives biomass accumulation by photosynthesis, partitioning of biomass between light-gathering and structural components of the plants, and an aging function. A hyperbolic relationship between plant nutrient uptake and biomass yield is assumed, and is confirmed for the mineral elements nitrogen (N), phosphorus (P), and potassium (K). It is concluded that the rate limiting process in the system is biomass accumulation by photosynthesis and that nutrient accumulation occurs in virtual equilibrium with biomass accumulation.

## Introduction

In a recent publication the authors discussed a model of yield response of corn to plant population and absorption of solar energy within the plant canopy [1]. Data from three field studies formed the empirical foundation for the mathematical model. The simple exponential model contained two parameters: one for upper limit on yield at high plant population and an exponential response coefficient. The model described the data very well and exhibited similarities among the three studies. In a textbook the authors have discussed various aspects of crop growth and yield [2], including a mathematical model of crop growth with calendar time. The expanded growth model incorporates the three basic processes of an energy driving function, partitioning of biomass between light-gathering and structural components of the plants, and an aging function. This model is used in the present analysis.

A simplified theory of biomass production by photosynthesis has been published by the authors [3]. The theory incorporates basic principles from mathematics and physics and uses data from the literature for a warm-season perennial grass as an empirical base. The strategy follows the procedure of *emergence* as described by Robert Laughlin [4] which means that development of the theory is guided by measurement and observation. This approach examines behavior of a large assemblage of matter, in contrast to the classical reductionist approach which breaks a system into its smallest parts and then describes interactions among the parts.

Data from a field study at Florence, SC, USA are used to evaluate application of the model [5] for corn (*Zea mays* L.). Many field studies have been conducted on the growth of corn, noted as examples in references [6]–[11]. Key measurements of accumulation of plant biomass as well as the mineral elements nitrogen, phosphorus, and potassium were established in the 1948 study by Sayre [6]. Effect of applied N and P fertilization on biomass accumulation was evaluated by Bar-Yosef and Kafkafi [7]. Interactions of plant population and applied N on biomass accumulation were measured by Rhoads and Stanley [8]. Dependence of yield on measured evapotranspiration for three different soils was reported by Tolk and Howell [9]. An exponential relationship was clearly demonstrated. The present article is not intended as a general literature review of either mathematical models or field studies on crop growth. It describes concepts and procedures for the expanded growth model on high quality data from a field study with growth of corn.

## Methods

The first step is to define relevant quantities (variables and model parameters): *t* is calendar time (referenced to Jan. 1), wk; *Y* is biomass yield (dry matter), Mg ha^−1^; *N_u_* is plant nutrient uptake (N, P, or K), kg ha^−1^; *N_c_* = *N_u_*/*Y* is plant nutrient concentration (N, P, or K), g kg^−1^. A common reference time is used to facilitate comparison among various studies. The second step is to utilize a mathematical model which relates biomass accumulation to calendar time. For this purpose we adopt the expanded growth model discussed in Section 3.5 of Overman and Scholtz [2], which can be written as
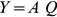
(1)where *A* is a yield factor, Mg ha^−1^; and *Q* is a dimensionless *growth quantifier*, defined by
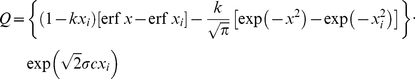
(2)in which the dimensionless time variable *x* is defined by
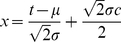
(3)with the parameters in Eq. (3) defined as 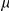 time to the mean of the solar energy distribution (referenced to Jan. 1 for the northern hemisphere), wk; 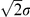 the time spread of the solar energy distribution, wk; *k* the partition coefficient between light-gathering and structural components of the plants, and *c* an aging coefficient for the plant species, wk^−1^. It follows that *x_i_* corresponds to the time of initiation of significant plant growth *t_i_*, wk. These parameters are discussed in more detail in the next section on application to the corn study at Florence, SC, USA. The ‘error function’, erf *x*, in Eq. (2) is defined by
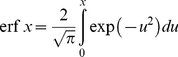
(4)with *u* as the variable of integration for the Gaussian distribution 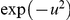. Values of the erf *x* can be obtained from a handbook of mathematical functions (*see Table 7.1* [12]).

Examination of data of coupling between plant nutrient accumulation *N_u_* and plant biomass *Y* leads to the hyperbolic phase relation
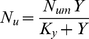
(5)with *N_um_* as potential maximum plant nutrient accumulation at high *Y* and *K_y_* is the value of *Y* at which *N_u_* = *N_um_*/2. This subject is explored in more detail in the next section of application to data from Florence, SC, USA.

## Results

**Figure 1:**
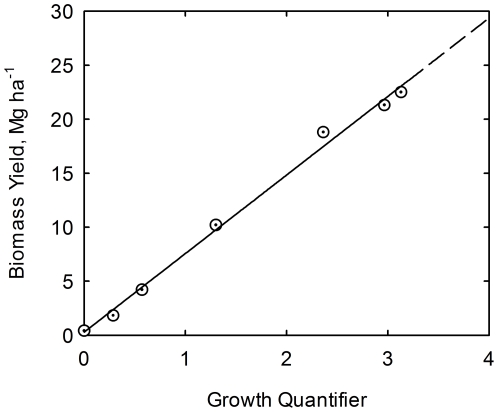
Correlation of biomass yield (*Y*) with growth quantifier (*Q*). Biomass yield data for corn at USDA research center at Florence, SC, USA [5]. Line is drawn from Eq. (8).

Data for this analysis are adapted from a field study by D.L. Karlen and associates [5] with ‘Pioneer 3382’ corn on Norfolk loamy fine sand (fine-loamy, siliceous, thermic Typic Paleudult) at the USDA-ARS Coastal Plains Soil, Water, and Plant Research Center at Florence, SC, USA. Data for 1982 and plant population density of 7 plants m^−2^ are used here. Planting date was April 2 (*t* = 15.0 wk). Fertilizer application was N-P-K = 268-36-224 kg ha^−1^. Data are given in Table 1 for each sampling time for calendar time *t*, biomass yield *Y*, and plant nutrient uptake and plant nutrient concentration for nitrogen, phosphorus, and potassium. Analysis of data from various studies has led to parameter estimates: 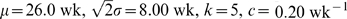. Now by varying the time of initiation, *x_i_*, it can be shown that maximum utilization of solar energy is obtained for *x_i_* = 0. This choice of the parameters leads to
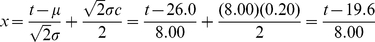
(6)It follows from Eq. (6) that *x_i_* = 0 corresponds to *t_i_* = 19.6 wk. Apparently a time interval of 4.6 weeks is required for germination of seeds and development of corn plants to reach optimum capture of solar energy and significant plant growth. Note that the effect of the aging function is to shift reference time from 26.0 wk to 19.6 wk. The growth quantifier equation becomes
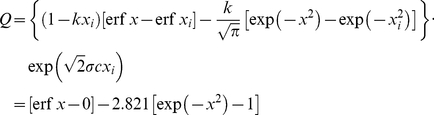
(7)Values adapted from the experiment are listed in Table 2. Linear regression of biomass yield on the growth quantifier leads to
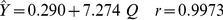
(8)where 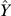 signifies an estimator of biomass yield *Y*. The quality of the correlation is confirmed in Figure 1. Equation (8) is in agreement with Eq. (1), the simple linear model. The next step is to explore the coupling between accumulation of plant nutrients and plant biomass. Equation (5) can be rearranged to the linear form
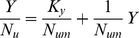
(9)Data for nitrogen, phosphorus, and potassium are now used to test the validity of Eq. (9). The value of *Y*/*N_u_* corresponding to each value of *Y* is calculated from Table 1. Linear regression then leads to

**Table 1:** Accumulation of biomass and mineral elements by corn at Florence, SC, USA.

*T*	*Y*	*N_u_*	*N_c_*	*P_u_*	*P_c_*	*K_u_*	*K_c_*
wk	Mg ha^−1^	kg ha^−1^	g kg^−1^	kg ha^−1^	g kg^−1^	kg ha^−1^	g kg^−1^
15.0	planting (April 2)						
19.6	0.406	13	32	1	2.5	18	44.2
21.0	1.83	60	32.8	6	3.3	118	64.5
22.0	4.21	110	26.1	13	3.1	219	52.1
24.0	10.2	171	16.8	22	2.2	298	29.2
26.8	18.8	198	10.5	34	1.6	267	12.5
29.5	22.5	207	9.2	34	1.5	293	13.0

**Table 2:** Correlation of biomass accumulation (*Y*) with the growth quantifier (*Q*) for corn at Florence, SC, USA.

*t*	*x*	erf *x*	exp (−*x* ^2^)	*Q*	*Y*
wk					Mg ha^−1^
19.6	0	0	1	0	0.406
21.0	0.175	0.1999	0.9698	0.285	1.83
22.0	0.300	0.3286	0.9139	0.571	4.21
24.0	0.550	0.5633	0.7390	1.300	10.2
26.8	0.900	0.7969	0.4449	2.363	18.8
28.8	1.150	0.8961	0.2665	2.965	21.3
29.5	1.2375	0.9198	0.2162	3.131	22.5
	∞	1	0	3.821	-----

**Figure 2:**
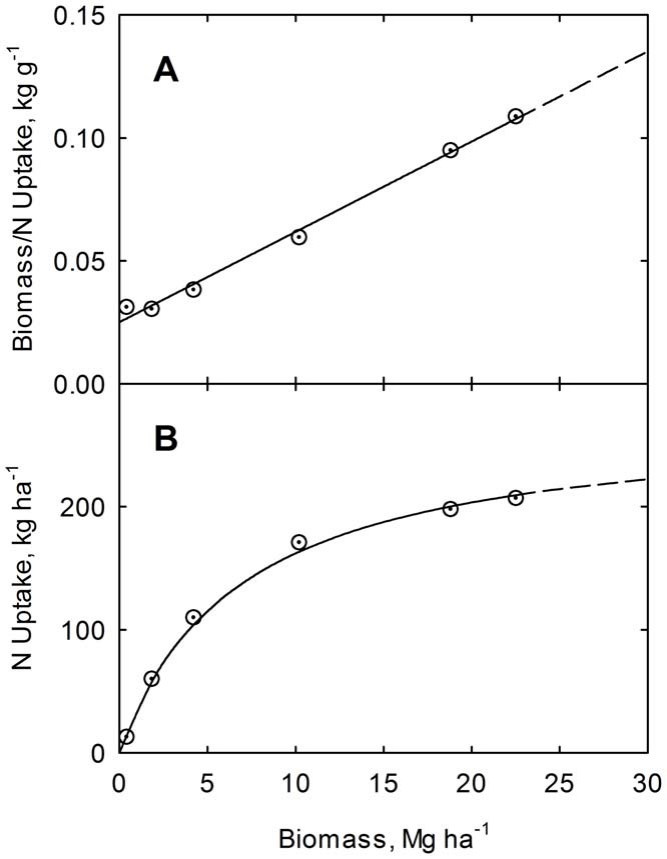
Correlation of plant biomass to nitrogen uptake ratio (A) and of plant nitrogen uptake (B) with biomass yield. Crop data for corn at USDA research center at Florence, SC, USA [5]. Line is drawn from Eq. (10). Curve is drawn from Eq. (11).

**Figure 3:**
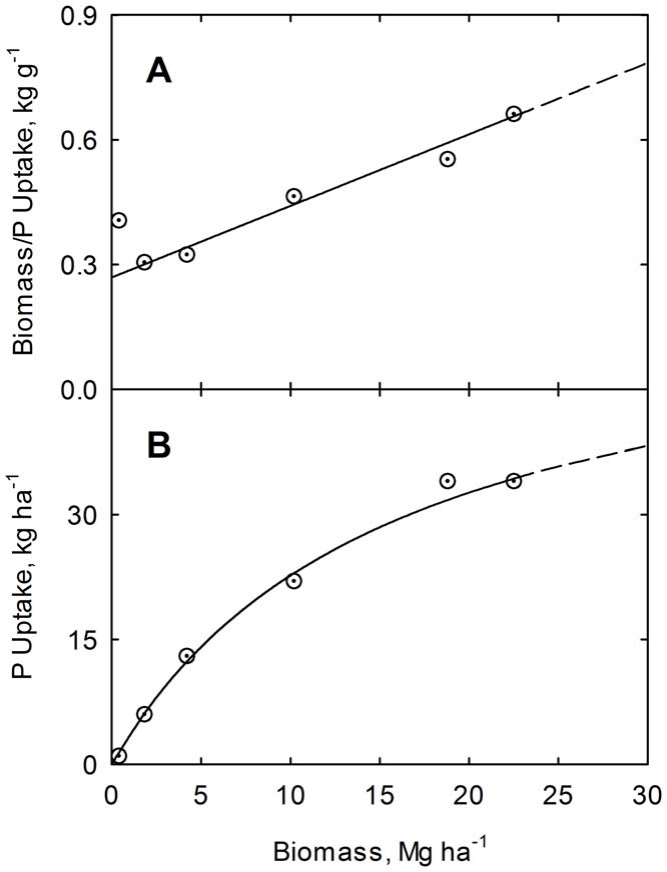
Correlation of plant biomass to phosphorus uptake ratio (A) and of plant phosphorus uptake (B) with biomass yield. Crop data for corn at USDA research center at Florence, SC, USA [5]. Line is drawn from Eq. (12). Curve is drawn from Eq. (13).

**Figure 4:**
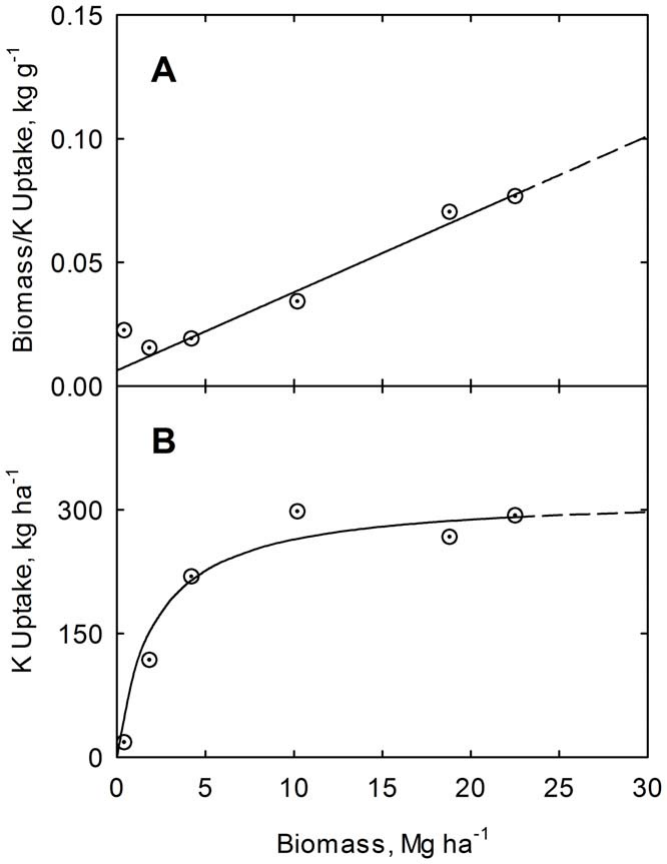
Correlation of plant biomass to potassium uptake ratio (A) and of plant potassium uptake (B) with biomass yield. Crop data for corn at USDA research center at Florence, SC, USA [5]. Line is drawn from Eq. (14). Curve is drawn from Eq. (15).

Nitrogen:
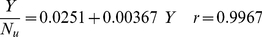
(10)

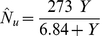
(11)Phosphorus:
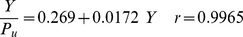
(12)

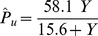
(13)Potassium:
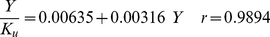
(14)

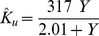
(15)Results are shown graphically in Figures 2, 3, and 4 for nitrogen, phosphorus, and potassium, respectively. The high correlations confirm the validity of the phase relations for this study.

## Discussion

**Figure 5:**
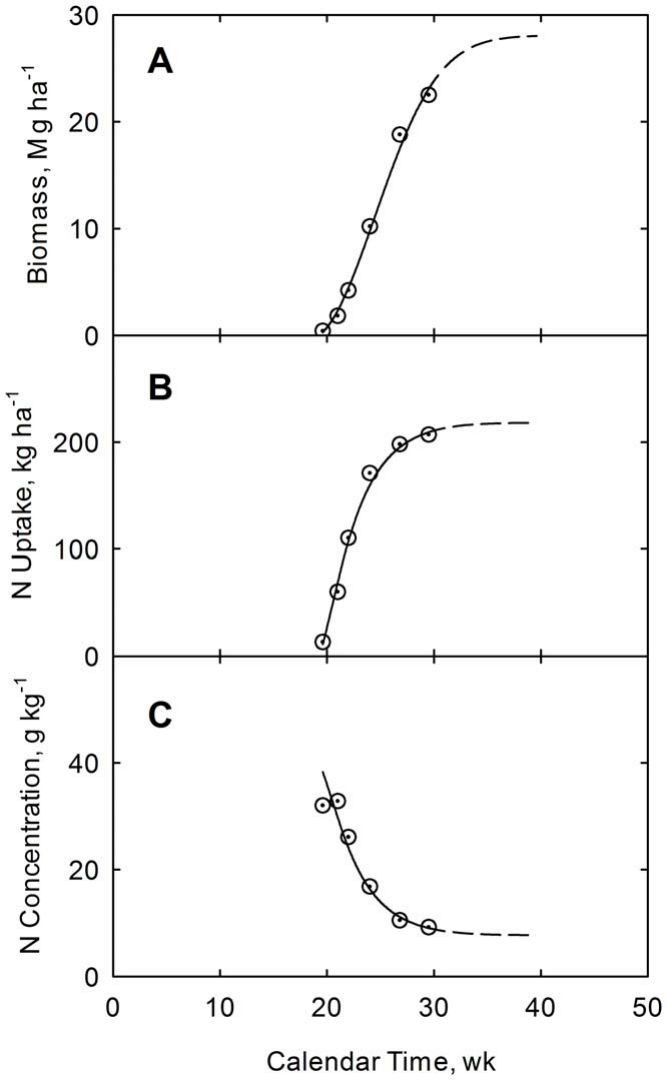
Accumulation of biomass yield (A), plant nitrogen uptake (B), and plant nitrogen concentration (C) with calendar time. Crop data for corn at USDA research center at Florence, SC, USA [5]. Curves are drawn from Eqs. (7), (8), and (11). (A).

The next step is to provide simulation of biomass (*Y*) and plant nitrogen (*N_u_*) with calendar time (*t*). Accumulation of the growth quantifier (*Q*) with calendar time follows from Eq. (7). Coupling of biomass yield with growth quantifier follows from Eq. (8). Coupling of plant nitrogen with biomass yield follows from Eq. (11). Coupling of plant nitrogen concentration is then defined by *N_c_* = *N_u_*/*Y*. Simulation curves are shown in Figure 5 along with values from the experiment. Close agreement between the estimated and measured values should be noted. The decline in plant nitrogen concentration with calendar time is explained by the shift from dominance of light-gathering (leaf) fraction with higher plant N in young plants toward dominance of structural (stalk) fraction with lower plant N in older plants. In cases where correlations are much lower than obtained in this analysis could signify either large scatter in the data and/or that the linear relationship is not valid. The high efficiency of nitrogen utilization by the plants in this study may be noted. Potential nitrogen uptake from Eq. (11) is 273 kg ha^−1^ for applied nitrogen of 268 kg ha^−1^, for an efficiency ratio of 273/268 = 1.02.

Numerous factors influence the value of the yield factor *A*. These include plant population, level of applied nutrients, water availability (such as rainfall or irrigation), and frequency of harvest (for perennial grasses). Some of these interactions have been detailed in Overman and Scholtz [1]&[2].

We can now interpret the meaning of this analysis. Both *Y* and *N_u_* are accumulating with calendar time and therefore represent rate processes. The rate limiting process in the system is biomass accumulation by photosynthesis. Phase relations (Eqs. (11), (13), and (15)) imply that accumulation of the mineral elements (N, P, and K) occur in virtual equilibrium with biomass accumulation. This conclusion is supported by the simplified theory of biomass production [3]. An excellent discussion of photosynthesis has been presented by Morton [13], with emphasis on what has been learned and what remains as open questions.

The expanded growth model is derived via classical methods. By making simplifying assumptions, an analytical solution can be found from linear differential equations that are based on key fundamental processes. Thus, the expanded growth model eliminates the need to use computer algorithms to solve the inherent complexity, and along with the nutrient accumulation phase relationship has been shown to closely agree with the data presented by Karlen et al. [5]. It is suggested that this procedure should be tested for other cases, including other crop species and experimental conditions, and other mineral elements (such as Ca, Mg, etc.). Some data suggest that the procedure utilizing nutrient accumulation phase relationship does apply for Ca and Mg [11]. The authors plan to examine the yields of light-gathering and structural components and the effect on nutrient accumulation using the expanded growth model in a future publication.
