# Correction: Accumulation of Biomass and Mineral Elements with Calendar Time by Corn: Application of the Expanded Growth Model

**DOI:** 10.1371/annotation/8fd75170-f1cd-4126-8d4a-3579712703f6

**Published:** 2012-01-27

**Authors:** Allen R. Overman, Richard V. Scholtz

There was an error in the funding statement. The correct funding statement is: “This analysis was funded by the Florida Agricultural Experiment Station. Publication of this article was funded by the University of Florida Open-Access Publishing Fund. The funders had no role in study design, data collection and analysis, decision to publish, or preparation of the manuscript.” 

